# Antigen-specificity measurements are the key to understanding T cell responses

**DOI:** 10.3389/fimmu.2023.1127470

**Published:** 2023-04-14

**Authors:** Rashmi Tippalagama, Leila Y. Chihab, Kendall Kearns, Sloan Lewis, Sudhasini Panda, Lisa Willemsen, Julie G. Burel, Cecilia S. Lindestam Arlehamn

**Affiliations:** Center for Infectious Disease and Vaccine Research, La Jolla Institute for Immunology, La Jolla, CA, United States

**Keywords:** T cell, antigen-specificity, TCR, sequencing, adaptive immunity

## Abstract

Antigen-specific T cells play a central role in the adaptive immune response and come in a wide range of phenotypes. T cell receptors (TCRs) mediate the antigen-specificities found in T cells. Importantly, high-throughput TCR sequencing provides a fingerprint which allows tracking of specific T cells and their clonal expansion in response to particular antigens. As a result, many studies have leveraged TCR sequencing in an attempt to elucidate the role of antigen-specific T cells in various contexts. Here, we discuss the published approaches to studying antigen-specific T cells and their specific TCR repertoire. Further, we discuss how these methods have been applied to study the TCR repertoire in various diseases in order to characterize the antigen-specific T cells involved in the immune control of disease.

## Setting the stage: T cell mediated immunity

The innate and adaptive arm of the immune system work in coordination to elicit host immune responses against a variety of pathogens. The adaptive response, a crucial component of the immune system, relies on antigen-specificity, and mediates action via B and T cells (1). These cells have a diverse and finely tuned repertoire of receptors with the ability to discriminate between self and non-self-antigens (2, 3). Upon antigen-specific activation individual T and B cells undergo clonal expansion to produce a population of identical antigen-reactive cells (4–6). While B cells are responsible for antibody mediated responses, T cells disseminate their action via a plethora of cell mediated responses (7). T cells are also critical effector cells for providing protection against wide range of pathogens and cancer, as well as maintaining self-tolerance. This broad range of functions is enabled by the diversity of T cell phenotypes and antigen specificities. T cell phenotypes range from highly cytotoxic effector cells to regulatory T cells that fight inflammation. These effector cells include the CD4 and CD8 lineage. Unlike CD4 T cells, which normally focus on protein antigens sourced from the extracellular environment, CD8 T cells preferentially identify antigens that are biosynthesized by infected or altered host cells. Both CD4 and CD8 T cells differentiate into independent memory cell lineages that release various and frequently mutually exclusive sets of cytokines in response to antigen contact and the proper co-stimulatory cues (8, 9). There are several different CD4 T helper (Th) subsets that have been found, but the Th1, Th2, and Th17 lineages are the most well-known. However, other CD4 Th cell subsets, such as the Th1*, especially in the context of mycobacterial infection (10–12), and Th9 and Th22 subsets that release IL-9 and IL-22, have become more well-known in recent years (13). The wide diversity of T cell phenotypes is a direct result of the wide repertoire of T cell receptors (TCR) and range of T cell antigen-specificities. In this review, we will be focusing on antigen-specificity in the context of T cells and their TCRs and discussing it in detail.

## Development of antigen-specific T cells

T cells are key mediators in mounting an effective cell mediated immune response. During T cell development, T cell precursors travel to the thymus, where they develop into mature T cells and are exported to the periphery where they can be activated by antigens and differentiate into effector and memory cells. T cell development is largely dependent on T cell receptor interactions which facilitates the transition of double positive T cell progenitors (CD4+ and CD8+) to single positive cells (CD4+ or CD8+) after thymal selection. During this selection, T cells bearing TCR with a high affinity for self-peptide Major Histocompatibility Complexes (MHC) undergo apoptosis (negative selection), whereas those bearing low-affinity TCR for self-peptides survive and differentiate into mature T cells (positive selection). This ensures that only those T cells that are self-tolerant survive while eliminating the self-reactive T cells. The T cells leaving the thymus are functional cells expressing unique and specific T-cell receptors (TCRs) that are both tolerant to self-antigens and restricted to self-MHC (14). These cells have the ability to responds to new antigens with a wide array of antigen specificities due to highly diverse TCRs. An immune response is initiated when naïve single positive T cells encounter processed antigen presented by antigen presenting cells (APCs). The TCR of naïve single positive T cells binds to the antigen-MHC complex which results in the proliferation and differentiation of antigen-specific effector cells that migrate to diverse sites and aid in pathogen clearance (15). The activated cells are short lived, although a subset of cells survive as memory T cell maintaining long term immunity (8, 16). It is very important to study these antigen-specific T cells as they play a central role in the adaptive immune response in various pathologies. In cancer, tumor-specific T cells are involved in effective anti-tumor immunity, whereas in infectious diseases, pathogen-specific T cells coordinate specific defense mechanisms. Apart from pathological contexts, antigen-specific T cells are also crucial for the formation of immunological memory (e.g., after vaccination) or for the maintenance of tolerance to self-antigens. The various applications of antigen-specific T cells and their TCRs will also be discussed in detail in this review.

## The role of TCRs in antigen-specific diversity in disease

T cells provide protection by recognizing processed antigens presented on Major Histocompatibility (MHC) molecules using its highly specific TCR. The ensemble of TCRs that are present within an individual at a particular time is called the TCR repertoire which are generated by the process of random recombination and selection. Each T cell expresses a unique TCR on its surface which facilitates antigen recognition and subsequent immune responses, as well as adding another dimension of variation within T cell populations.

The core TCR complex contains two TCR chains and six complementarity-determining regions (CDR). There are four TCR genes in the human genome; TCRα, TCRβ, TCRγ, and TCRδ. The majority of T cells express α and β isoforms to form a heterodimer i.e., αβ T cells or more generally referred to as just T cells. The α/β TCRs bind to antigenic peptides presented in molecular grooves on the surface of MHC I or MHC II molecules present on APCs (17–20). Only a small proportion of T cells express TCRγ and TCRδ isoforms known as γδ T cells. TCR chains are made up of an extracellular region, a transmembrane region and a short cytoplasmic tail. The extracellular region consists of a variable domain (V) that serves as antigen binding site and a constant domain (C) used for interaction with CD3 chains (21). The V domain typically has a marked sequence variation while the rest of the chain remains conserved. Each V domain has three Complementarity-determining regions (CDR). Various studies from the past few decades showed that the CDR1 and CDR2 regions of the TCR interact with MHC while the CDR3 region interacts with the antigenic peptide (22–25). The TCR is glycosylated, and recent studies have shown that the amount of glycosylation may vary depending on the level of T cell activation (26, 27). The generation of T cell diversity arises from genetic recombination of DNA encoded segments by combinatorial somatic V (variable), D (diversity) and J (joining) recombination using RAG1 and RAG2 recombinases. The α chain is generated by VJ recombination while the β chain is generated by VDJ recombination. Theoretically, a TCR repertoire consists of 2x10^19^ unique TCRαβ pairs generated by the recombination process along with non-templated addition or deletion of nucleotides between spliced gene segments. Therefore, each T cell repertoire is shaped by both genetically determined biases, as well as immune exposures. However, the actual diversity is likely lower, which can potentially be explained by the selection process and number of T cells present. However, it has not yet been thoroughly investigated how much genetic background of an individual influences the diversity of their TCR repertoire.

In recent years, high-throughput TCR profiling has been widely used to define the interaction between TCRs and the matching peptide/MHC complexes. A TCR sequence can be used as a unique identifier of T cell clones and can be used for measuring antigen-driven clonal expansion of T cells. Characterizing the TCR repertoire can describe T cell dynamics in a wide range of diseases, including malignancies, autoimmune disorders, and infectious diseases. With the help of TCR sequencing, some studies have demonstrated that T cell populations change after numerous immunization cycles or pathogen exposure (28, 29). This change is crucial for the understanding of disease pathology and when designing therapeutic strategies. Studying the TCR diversity in patients across time can also shed light on the kinetics of T cell clones that may correlate with clinically relevant features or ongoing treatment. In this review, we highlight both TCR-sequence dependent and independent techniques of identifying antigen-specificity *ex vivo* and *in vitro* and their potential applications in different diseases.

## Antigen-specific T cells can be identified in several ways

Antigen-specificity of a T cell can be deciphered directly using peptide-MHC (pMHC) multimers, and/or indirectly using surrogate markers of antigen-specificity.

### Peptide-MHC multimers are the gold standard for identifying antigen-specific T cells

The gold standard technique to identify antigen-specific T cells is the use of peptide-MHC (pMHC) multimers, which are stable complexes of several identical pMHC monomers that can specifically bind to a given TCR (30, 31). The seminal and still most commonly used scaffold type is a tetramer (i.e., containing 4 pMHC) (30), but other multimers exist such as dimers, pentamers and dextramers (32, 33). The pMHC multimers are typically conjugated to a fluorochrome, and thus enable for direct visualization and isolation of antigen-specific T cells using flow cytometry. The pMHC multimers can be used for the study of both MHC-I restricted (CD8) and MHC-II restricted (CD4) T cell populations. Their use is more prevalent for the analysis of antigen-specific CD8 T cells, as pMHC-I multimers are associated with greater TCR affinity compared to pMHC-II multimers (34), and epitope/MHC restriction prediction tools generally perform better for MHC-I than for MHC-II epitopes (35). A myriad of studies, that have been reviewed previously (36–38), has used pMHC multimers for identifying and monitoring the frequency, phenotype and function of antigen-specific T cells in the context of infectious diseases, vaccination, auto-immunity, allergy, and cancer.

The main advantage of pMHC multimers is their usability to identify antigen-specific T cells *ex vivo*. Thus, unlike assays requiring *in vitro* stimulation that will significantly affect the cell transcriptome and phenotype, tetramers can give information on the phenotype of antigen-specific T cells *in vivo* at the time of sampling. Additionally, since pMHC multimers directly bind antigen-specific T cells, the risk of contamination with bystander activated T cells is lower compared to indirect antigen-specific T cell profiling techniques, for instance those based on the production of pro-inflammatory cytokines after *in vitro* stimulation (39). Lastly, a key advantage of pMHC multimers is that they can be used without the presence of Antigen presenting cells (APC), for instance on T cell clones derived from tumor infiltrating lymphocytes or TCR transduced T cell lines (40). The major limitation of pMHC multimers is that the exact epitope sequence and its associated MHC restriction are required for each individual antigen-specific T cell population targeted to study, limiting their application to already well-characterized epitopes. Another limitation of pMHC multimers is that the frequency of binding T cells is generally very low, and therefore requires analysis of large sample volumes. Moreover, due to low sensitivity, staining protocols need to be carefully optimized for each pMHC multimer to achieve maximum specificity and sensitivity (40–42).

**Figure 1:**
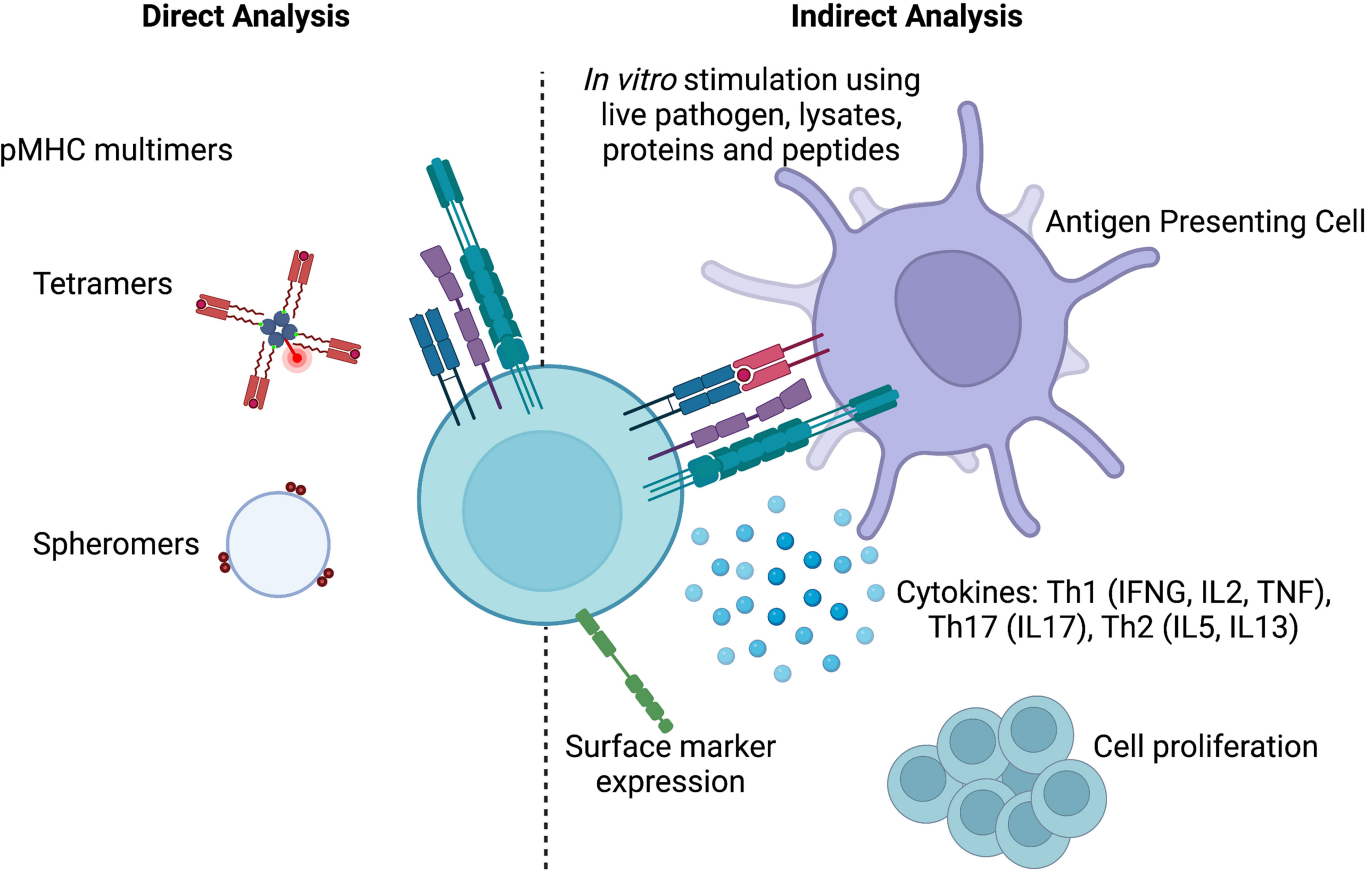
Methods to identify antigen-specificity. pMHC multimers allows the direct identification of antigen-specific T cells, while indirect methods rely on cytokine production, surface marker expression or cell proliferation upon *in vitro* stimulation. Created with BioRender.com.

Since their first application in 1996, significant advances have been made to tackle the limitations of pMHC multimers. New technologies use heavy metal or DNA labelled pMHC multimers that enable the simultaneous interrogation of hundreds to thousands of epitope specificities in a single sample at the single-cell level (43, 44). More recently, the development of spheromers, a multivalent self-assembly system that simultaneously displays six pMHC dimers, allows for the detection of antigen-specific CD4 and CD8 T cells at much greater specificity and sensitivity compared to their tetramer and dextramers counterparts (45) (**Figure 1**). Furthermore, the development of MHC epitope prediction tools, as well as community-based epitope databases such as the Immune Epitope Database (IEDB) (46), were instrumental to identify new epitope/MHC combinations that can be used for multimers. More recently, the discovery of non-classical T cells and their ligands has initiated the development of non-classical MHC multimers tailored for the study of antigen-specific NKT, γδT or MAIT cells (47). Despite all these advances, it remains questionable whether the pMHC multimer technology will ever be sufficient to create a complete list of all classically restricted epitopes and ligands of non-conventional T cells that are being recognized in the global human population.

In conclusion, pMHC multimers can be used to directly label antigen-specific T cells and bypass the need for *in vitro* stimulation. Due to their limitations in design and optimal use, they are best suited for studies restricted to a few well-characterized antigen-specific T cell populations.

### Using surrogate markers of antigen specificity bypasses the need for tetramers

Indirect analysis of T cell antigen specificity requires *in vitro* stimulation to measure specific T cell features. Most notable/commonly used features are cytokine production, proliferation and the expression of activation induced markers (AIM) (**Figure 1**). The complexity and duration of *in vitro* stimuli used in these assays vary from single peptides, multiepitope pools (so called “megapools”) to whole pathogen preparations that can last from a few hours to several days. Longer stimulations are preferred when the aim is to enrich cell populations that are low in numbers, while shorter stimulations limit the possibility of bystander activation.

The most commonly used cytokine to define antigen-specificity is IFN-γ. Along with TNF-α and IL2, it is typically used to define Th1 polarized antigen-specific T cells. However, using different cytokine combinations, many other T helper cell subsets can be defined, such as Th2 (producing IL-4, IL-5, and IL-13) or Th17 (producing IFN-γ, IL-17) cells. The two main methods to measure cytokine production after antigen-specific stimulation are ELISpot/FluoroSpot and intracellular cytokine staining (ICS).

The ELISpot assay is highly validated (48) and currently one of the most frequently used assays (49) to detect and quantify antigen-specific T cell responses in peripheral blood in clinical trials (50). Whereas these assays were originally designed to capture only one cytokine, the FluoroSpot technology can simultaneously measure up to four cytokines and this number is likely to increase in the future (51, 52). ELISpot/FluoroSpot assays are powerful due to is their high sensitivity. Less than 100 cytokine molecules can be detected and the lower detection limit can be less than 10 cytokine-producing cells per million cells (53). Their major limitation is the inability to identify/phenotype the exact cells responsible for the response.

In ICS, antigen-stimulated cells are fixed and stained with intracellular fluorescently labeled cytokine-targeting antibodies and subsequently analyzed via flow cytometry (54, 55). The main advantage of ICS is that cytokine production can be measured per cell. This method allows for the quantification of the cells that produce the targeted cytokine/s (56), as well as deducing their exact phenotype (e.g., memory/naïve phenotype). The main disadvantage of the ICS is that it requires cell fixation, which can preclude downstream analysis. Cytokine-based assays are thus easily performed and are ideal for routine analysis in many clinical trials. They can be used to study antigen-specific T cell populations with a well-defined cytokine polarization profile.

Another widely used technique for capturing antigen-specificity is through cell proliferation assays. Typically, these assays use cell proliferation dyes, such as CFSE or Cell Trace Violet, to fluorescently label all cells prior to stimulation (57, 58). Upon division, each daughter cell will receive half of the fluorescence intensity from the original cell which enables tracking the number of divisions each cell has undergone via flow cytometry. Similar to ICS, this assay offers single-cell resolution and can be used to determine not only the frequency but also the phenotype of dividing cells. This assay is also particularly well suited for the identification of rare T cell populations, such as allergen- (59) or autoantigen- (60) specific T cells. Proliferation dyes are limited by their toxicity and requires careful protocol optimization to obtain a stable and homogenous fluorescence intensity in the originally labelled cell population. Dying cells also lose fluorescence intensity, but newer versions that overcome these challenges are constantly emerging (58, 61).

The co-expression of surface protein activation markers upon *in vitro* stimulation is measured via flow cytometry and used to identify antigen-specific T cells (62, 63). Well defined activation markers are: CD20, CD25, CD40L, CD69, CD107a, CD137, CCR7 CXCR5, OX40, PD-1, and PD-L1. Unlike cytokine assays, AIM assays are not limited to a handful of cytokines, and are also more sensitive. Using the AIM assay, Bowyer et al. detected more antigen-specific T cells compared to ELISpot and ICS with comparable (CD4+) or even lower (CD8+) background signal (64). AIM assays have been successfully applied to identify antigen-specific T cells in the context of infection and vaccination (65–67) and their popularity is increasingly growing. A variation of AIM is the ARTE (Antigen Reactive T cell Enrichment) assay. Upon interaction with APCs, CD154 expression peaks at 6-8 hours on CD4 T cells and are captured by magnetic enrichment prior to stimulation. This method provides a reliably way to isolate rare populations of T cells thus making it a popular choice for studying antigen-specificity (68–71).

### TCR-sequence based identification of antigen-specific T cells

The TCR is composed of two different chains, each containing variable and constant regions. The CDR3 region of each chain is associated with the highest number of recombination events, and is directly in contact with the epitope, thus critical for antigen recognition. A given TCR sequence will be shared by all cells originating from the same T cell, and all T cells from that clone will share the same antigen specificity. Thus, the association of TCR sequences to antigen specificity could, in theory, be used to analyze the entire antigen-specific T cell repertoire in a given individual, without prior knowledge of the MHC sequence/restriction (as needed with tetramers). Additionally, TCR sequences could be used to trace the fate of clones over time and pre-post stimulation. This technique could be applied on longitudinal samples of a disease cohort undergoing treatment, pre-post vaccination, and also before and after *in vitro* stimulation (discussed below). In this section, we review the current techniques of TCR sequencing and how it can be linked to antigen-specificity in T cells.

### TCR sequencing techniques can be either direct or indirect depending on the context

TCR sequencing techniques can be broadly divided into two categories: i) direct techniques that perform targeted sequencing of the TCR, and ii) indirect techniques that employ bioinformatic algorithms to derive TCR sequences or specificities from bulk or single-cell RNA sequencing data.

Targeted TCR sequencing is typically done using first a targeted amplification step with specific primers spanning the CDR3 region (72, 73). For targeted TCR sequencing, the starting material can be either genomic DNA or cDNA depending on the context and has its own pros and cons (74). The first TCR repertoire analyses were done using bulk TCR sequencing. Pioneer studies include the ones from Freeman et al. (75) and Robins et al. (76), which identified tens of thousands of distinct TCR beta chain sequences in the peripheral blood of healthy individuals, revealed a higher repertoire diversity than previously expected. With rapid technological advances, targeted TCR sequencing can now be performed at the single-cell level, in combination with single-cell RNA sequencing (72, 73). It is even possible to get the entire TCR repertoire of a given sample at the single-cell level, in one single experiment (77). The advantage of bulk over single-cell TCR sequencing is that it is less expensive, and can be done on a larger number of cells, so it is a great option for mining the entire TCR repertoire of a given sample. Conversely, the advantage of single-cell TCR sequencing is that it offers chain pairing, and can provide functional information on a given TCR cell clone (e.g., transcriptome).

Since TCR αβT cells represent the majority of T cells in humans, and have been extensively studied for their MHC/peptide mediated antigen-specificity, the vast majority of TCR sequencing tools available are tailored for their use. Bulk TCR sequencing is almost exclusively done on the TCRβ-chain, as it contains a higher diversity compared to the α-chain (78). More recently, novel tools have been developed for the study of the TCR repertoire of non-conventional T cells. For instance, using custom designed primers, direct sequencing of TCRγ and TCRδ chains from γδT cells can be done at both bulk (79) and single-cell level (80). The study of antigen-specific TCRs in non-conventional T cells is reviewed in more detail in the following sections.

Repertoire sequencing can be expensive and consumes samples that may be available in limited supply. Repertoire construction tools offer an alternative to this problem by mining RNA-seq data for TCR and BCR sequences. Algorithms that can infer the full-length sequence of the immune receptor are preferred as it can facilitate better receptor-antigen interaction modeling. However, computational methods such as V’DJer (81), MiXCR (82), CATT (83) and ImRep (84) can only reconstruct CDR3, and are thus limited in their use to assemble full-length V(D)J receptor sequences. For single-cell platforms such as SMART-seq, pre-existing tools such as BALDR (85), BASIC (86), and VDJPuzzle (87), and even MiXCR have now been developed to construct full-length paired TCR or BCRs (88). Another tool that has gained popularity recently is TRUST4 which is a redesign of the TRUST algorithm but with substantially enhanced features and performance for αβ/γδ T cells and B cells from both bulk and single-cell RNA-seq (89). TRUST4 has proved to be far superior to other comparable methods such as MiXCR and CATT in both performance, sensitivity and speed.

### TCR sequences can be linked to antigen-specificity

Both direct and indirect techniques produce TCR sequences which can be used in conjunction with other methods to infer antigen-specificity either at bulk or single-cell level. For e.g., cells expressing AIM markers could be RNA sequenced and their respective TCR repertoires can be generated for downstream analysis. This is a powerful method to associate both cellular phenotypes with specific TCR sequences and thus can shed light on clonality of antigen-specific T cells. In this section we discuss how this can be achieved using both experimental and/or computational methods (**Table 1**).

**Table 1:** Experimental and computational methods for linking TCR sequences to antigen-specificity.

Method	Category	Summary	Strenghts	References
Antigen-specific *in vitro* expansion	Experimental	*In vitro* culture with antigens of interest for 14 days, followed by bulk TCR sequencing.	Can be applied to a wide range of antigens. No need to know the exact epitope sequences and their associated MHC restriction.	90, **Figure 2**
Tet-seq	Experimental	Single-cell sequencing of tetramer-stained cells using fluorescently labeled, DNA-barcoded pMHC tetramers.	Suitable for isolation of rare cells.	91, 92
MATE-seq	Experimental	Single-cell analysis of tetramer-stained cells using magnetic nanoparticle-barcoded pMHC tetramers linked to photocleavable TCR-specific primers to capture both TCR sequence and antigen-identity within a single cell.	Suitable for isolation of rare cells.	93
ENTER-seq	Experimental	Engineered lentiviruses to capture TCR-pMHC combinatorial interactions.	Higher sensitivity than tetramers due to the ability of lentiviruses to display a higher number of pMHC molecules on the cell surface. Does not require the synthesis of individual peptides to be loaded onto pMHC molecules.	94
Reverse phenotyping	Experimental	Single-cell sequencing before and after stimulation with antigens of interest. The TCRs specifically expanded after antigen-specific stimulation can be used as a barcode to identify antigen-specific cells before stimulation.	Identifies the *ex vivo* phenotype of antigen-specific T cells without the use of tetramers	95
TIL co-cultured with APCs	Experimental	Tumor infiltrating lymphocytes (TIL) are co-cultured with tandem minigene transfected or peptide pulsed autologous APCs before single-cell sequencing	Suitable to identify neoantigen-specific TCRs in a high-throughput manner for clinical applications	96, 97
ImmunoMap	Computational	Sequence alignment approach for assessing global similarities and relies on PAM10 matrix	Allows an intuitive appreciation of TCR repertoire characteristics that reconciles the structure and function of the repertoire.	98
TCRdist	Computational	Determines similarity between CDR regions by calculating weighted mismatch distance using alignment with BLOSUM62 substitution matrix.	First specialized single-cell TCR similarity measure combining both alpha and beta chains.	99
CDRdist	Computational	Uses a similar approach to TCRDist but only takes CDR3 sequences into account using local alignment and the BLOSUM45 substitution matrix.	Generates longer matching substrings in alignment allowing for a larger physico-chemical diversity.	100, 101
GLIPH2	Computational	Combines both global similarity metrics and local amino acid motifs to cluster TCRs and predict their HLA restriction.	Helps reduce noise by focusing only on short amino acid motifs within larger TCR sequences.	24, 102
TCRMatch	Computational	Matches TCR beta chain CDR3 sequences against the existing sequences in the IEDB to identify antigen specificity (and associated HLA restriction) of each hit.	Available as a web server tool, constantly updated with the ever-growing IEDB database	103
NetTCR-2.0/2.1	Computational	Utilizes a complex convolutional neural network (CNN) to predict TCR-pMHC interactions based on the amino acid sequences of the peptide and CDR3 region of the TCR chains.	CNNs can learn sequence motifs through training and thus perform well on datasets similar to training dataset.	104
DeepTCR	Computational	DeepTCR is a platform for both supervised and unsupervised deep learning that can be applied at both the individual TCR level and repertoire level	Same advantages as NetTCR-2.0	105
TCRAI	Computational	TCRAI utilizes a similar neural network as DeepTCR.	The flexible architecture, ID convolutions, batch normalization of CDR3 sequences and lower dimensional representations for the genes allows TCRAI to learn stronger gene associations making it a stronger performer compared to its rival DeepTCR.	106

### Experimental techniques of identifying antigen-specific T cells

**Figure 2:**
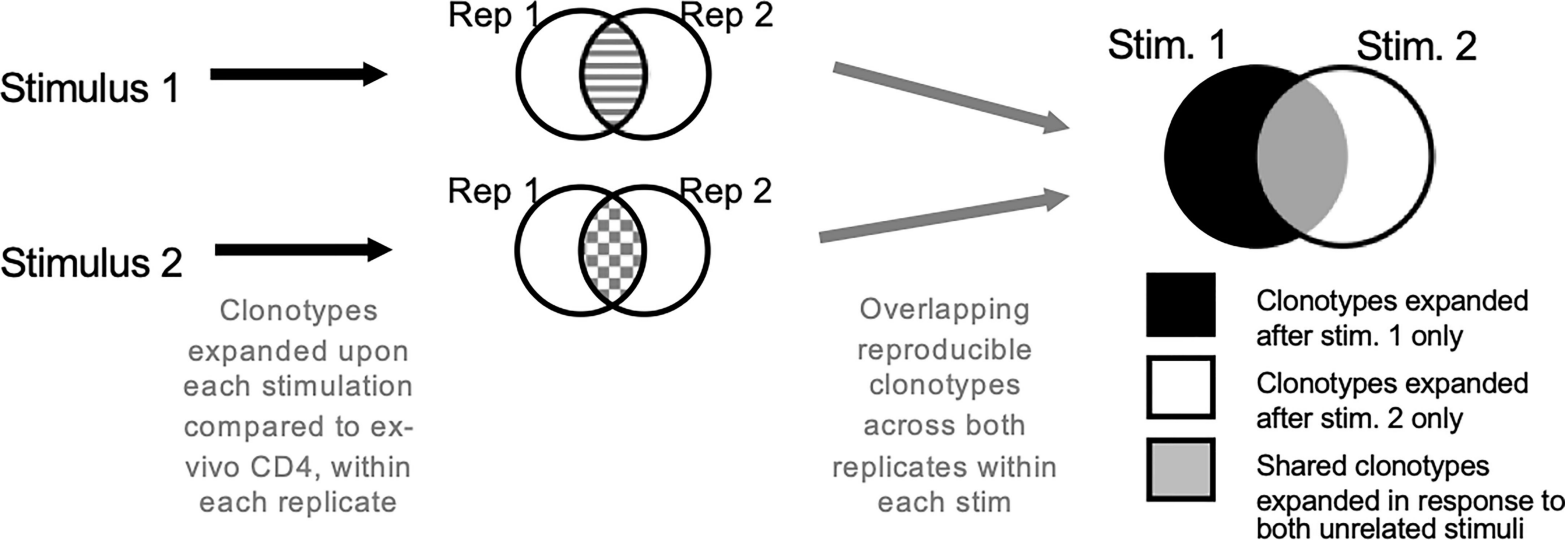
Antigen-specific TCR repertoire using *in vitro* culture. Cells are stimulated with antigens or epitope pools. Only those clonotypes that expanded upon stimulation compared to *ex vivo* T cell samples across both replicates are analyzed further. The clonotypes are overlapped to obtain antigen-specific clonotypes. Clonotypes that expand in response to multiple unrelated stimuli are excluded.

A relatively straight-forward technique to determine the antigen-specific TCR repertoire is using *in vitro* cultures to investigate the specific expansion of antigen-reactive clonotypes (90). This method used by our group has successfully determined both vaccine-antigen and auto-antigen specific TCRs (90) (**Figure 2**). PBMCs were stimulated with antigens of interest for 14-days *in vitro* after which the TCR repertoire was determined. Statistical variation was controlled for by culture replicates. Bystander activated cells and/or cells already activated by antigens before the sample was taken was controlled for by including an unrelated antigen and excluding TCRs that are expanded under multiple conditions. To determine which TCRs that expanded the productive repertoire was compared to an *ex vivo* sample of T cells from the same participant. This allowed identification of the TCRs that were antigen-specific, and subsequent computational analysis of public clonotypes and clonotype groups (as described below). We have implemented this method at the single-cell level in a group of TB patients undergoing treatment to isolate *ex vivo* antigen-specificity. Our goal is to 1) identify all cell phenotypes associated with antigen-specificity and 2) trace their fate from diagnosis/pre-treatment to treatment success. For *in vitro* stimulation component we employed megapools, which are synthetic peptides designed to carry proteins or epitopes of interest (107–111). They can be selected based on MHC binding to target either CD4 or CD8 T cell responses (35, 112). The advantage of megapools is the high rate of reproducibility of results due to limited variation between batches. Our group designed a Mtb-specific peptide pool of 300 MHC class II restricted epitopes (MTB300) which we and others have validated (113–116) for this purpose. Due to the overlap of epitopes recognized by MHC of multiple species, MTB300 has shown to capture T cell reactivity in mice and non-human primates, attesting to its versatility (115, 117–120).

Combining tetramer technology with single-cell sequencing has enabled the interrogation of antigen-specificity directly *ex vivo*. Tetramer-associated TCR sequencing (tetTCR-seq) allows to simultaneously profile TCR sequences, cognate antigen specificities, gene and surface protein expression at the single-cell level (91, 92). As highlighted by Zhang et al., large library of fluorescently labeled, DNA-barcoded pMHC tetramers were constructed using *in vitro* transcription/translation (IVTT). Next, tetramer-stained cells were single-cell sorted and the DNA-BC and TCR αβ genes were amplified by RT-PCR. A molecular identifier was included in the DNA-barcode to provide absolute counting of the copy number for each species of tetramers bound to the cell. Finally, nucleotide-based cell barcodes were used to link multiple peptide specificities with their bound TCRαβ sequences. DNA-barcoded pMHC tetramers were compatible with isolation of rare antigen-binding precursor T cells (121), making tetTCR-Seq a versatile platform to analyze both clonally expanded and precursor T cells.

Microfluidic techniques such as MATE-seq uses magnetic nanoparticle-barcoded pMHC tetramers linked to photocleavable TCR-specific primers to capture both TCR sequence and antigen-identity within droplets (93). T cells are incubated with a library of nanoparticle-barcoded pMHCs and purified magnetically. The single cells are captured as droplets and lysed, and the nanoparticle-barcoded pMHCs are exposed to UV light, releasing RT-PCR primers targeting TCR αβ C regions. Because these primers are linked to a DNA barcode corresponding to the pMHC, the TCR sequence and antigen specificity can be coupled at the single-cell level even after pooling and sequencing. There are two major limitations to this method. 1) This method is limited to a few pMHC tetramers due to limitations in library construction, 2) It can only be applied to T cells with known antigen-specificities (72).

ENTER-seq (lentiviral-mediated cell entry by engineered ligand-receptor interaction) uses engineered lentiviruses at multiple levels to systematically deorphanize TCR-pMHC interactions. GFP fused viruses with single chain of MHC infused with beta 2 microglobulin and covalently linked peptides are used to determine if the viruses can specifically bind to target cells (94). While this method is theoretically similar to DNA-barcoded library of pMHC tetramers, ENTER-seq has several advantages (122). 1) Libraries can be prepared parallelly by DNA synthesis, and thus does not require the synthesis of individual peptides to be loaded on to the pMHC molecules. 2) By leveraging lentivirus biology there is more uniform barcode oligonucleotide loading during the conjugation reaction (123). 3) ENTER-seq can be more sensitive due to the ability of the virus to display more envelope proteins per viral particle unlike pMHC tetramers which are four linked molecules by definition (124).

While tetramers are preferred, they are limited by their use as described in previous sections. There are ways to overcome these challenges and bypassing the use of tetramers when interrogating antigen-specificity. This method requires samples to be sequenced before and after stimulation. The TCRs present before and after can be used as a barcode to link antigen-specificity and to reverse phenotype the targeted cells (95). Using this method Fischer et al., found antigen-reactive clonotypes and they validated reactive TCRs by transgenic T cells using CRISPR/Cas9-mediated OTR. Furthermore using *in vitro* stimulation, they were able to decipher states of T cell activation/reactivity and associated it with severe or mild disease.

In another method, tumor infiltrating lymphocytes were co-cultured with tandem minigene transfected or peptide pulsed autologous APCs before single-cell RNA-seq. Multiple TCR sequences associated with cells expressing high levels of IFN-γ and IL-2 were identified (96). The identified TCRs were transduced into donor T cells using cloned retroviral vectors, and these transduced cells were able to specifically recognize neoantigens present by autologous APCs (97). This approach is an efficient procedure to isolate neoantigen-specific TCRs for clinical applications and basic translational research.

Unlike with tetramers, single cell sequencing after differential antigen stimulation offers the ability to identify and characterize antigen-reactive T cells by their states of reactivity which is not always possible with multimers. It thereby contributes to understanding the adaptive immune response which will provide a guide to enhance and accelerate development of therapies and vaccines for existing and emerging pathogens.

### Computational methods of deducing antigen-specificity

Another challenge of identifying antigen-specificity is determining which antigens are recognized by a particular TCR. Computational methods of epitope prediction are relatively new (23, 125–127) and it involves training a supervised machine learning model on TCR-antigen pairs to classify and predict antigen specificities of unknown TCR sequences. Unfortunately, the accuracy of the prediction rate on full TCR repertoires are low (23, 125) and models must be trained separately on different epitopes or sets of epitopes. Despite the advances in computationally driven algorithms for better epitope prediction, antigen-specific responses are complicated by cross-reactive TCRs which interfere with precise linking of TCR to biological function (128, 129). Thus, epitope prediction models require an experimental validation method to determine TCR specificity.

Algorithms which cluster TCRs, exploit similarity between TCRs with the aim to identify antigen-specificity. This works with the assumption that TCRs belonging to a specific group should recognize the same pMHC and this is achieved in two ways. 1) comparing global similarities across whole TCRs or CDR3 regions, 2) local similarities focusing on small amino acid motifs. ImmunoMap algorithm is an example of sequence alignment approach for assessing global similarities and relies on PAM10 matrix, large gap penalties and hierarchical clustering to group similar CDR3s (98). TCRdist was developed as a more focused approach to cluster TCRs based on a distance-based metric on both α and β chain of the receptor (99). It is a similarity weighted mismatch distance using alignment with BLOSUM62 substitution matrix to calculate similarity between CDR regions (100). Gap penalties are assigned to CDRs based on conserved short length motifs. Generally, gap penalties are low for CDR1 and CDR2, but increase for CDR3 as it is responsible for binding. Distance between two TCRs is calculated by summing over scores for each CDR on both chains, as well as an additional variable loop (CDR2.5). The CDR3 loop scores on both chains is upweighted in the sum, and TCRs are clustered using TCR distance. TCRdist is the first specialized single-cell TCR similarity measure which combined both α and β chains. However, it must be noted that this metric has not been evaluated on complex repertoires originating from responses from multiple epitopes. CDRdist uses a similar approach but only takes CDR3 sequences into account using local alignment and a substantial gap penalty with BLOSUM45 (100, 101). This combination generates longer matching substrings in alignment allowing for a larger physico-chemical diversity.

An alternative to the scoring approaches described above is to identify short motifs within TCR sequences. The underlying hypothesis of this approach is hot spot interaction, which states that only short stretches of similar amino acid residues are responsible for epitope binding (130–132). Thus, using short stretches of amino acids of length k (k-mers) to evaluate TCR receptor similarity could help reduce noise that is generally associated with using entire sequences. K-mers allows researchers to pinpoint dominant motifs driving TCR-epitope specificity rather than expanded clones. Thomas et al, demonstrates this on murine CD4 T cells following *M. tuberculosis* immunization (133, 134). Every CDRβ3 sequence was encoded as k-mers of length 3, and each triplet was encoded as a set of Atchley factors that corresponded to its physico-chemical properties (135). The authors generated a code book that reduced the set of representative triplets to describe the complete pooled dataset. This is done by pooling and subsampling triplets from all samples, and grouping them by k means clustering. A single representative triplet is selected to represent each cluster. Each murine repertoire is assigned a triplet vector based on the most similar triplet in the codebook. The repertoire representation is converted into a feature vector that is used for hierarchical clustering and Support Vector Machine (SVM) analysis. Both these methods could distinguish between immunized and non-immunized mice, but time points following immunization were not distinguishable. A major finding from this study is that the results strengthen the importance of TCR repertoire diversity as many private TCRs contribute to T cell responses to the same antigen in generically identical mice.

GLIPH (Grouping of Lymphocyte Interactions by Paratope Hotspots) combines both global similarity metrics and local amino acid motifs to cluster TCRs and predict their HLA restriction (24, 102). One study evaluated the efficiency of GLIPH by using publicly available CDR3 with known specificities, as well their own pMHC tetramer sorted human CD4 and CD8 data (24). Using GLIPH they searched for enriched conserved TCR motifs of length 2, 3 and 4 within TCR multimer repertoires in the CDRβ3 region. The distance metric was calculated by combining global and local TCR sequence similarity, V gene usage, CDR3 length bias, structural peptide antigen contact propensity and other features. GLIPH grouped 94% of the clustered TCRs together with others of the same specificity. In another evaluation with CD4 Mtb-specific T cells from 22 individuals with LTBI, showed that enrichment of motifs can organize TCRs within or across individuals. The authors state that GLIPH can be used independently of knowing epitope specificity. A major drawback of GLIPH was that it lost efficiency and accuracy when analyzing >10,000 TCRs. GLIPH2 was designed to process millions of TCR sequences overcoming this challenge (102).

There is no one single tool that outperforms the rest in its ability to classify TCR repertoire specificity. Biology is not simple and complexities such as cross reactive TCRs that bind to multiple antigens introduce challenges to computational models. TCR binding in itself is not sufficient to elicit a T cell response, and these methods do not take into account binding affinity, stability, co-stimulatory signals that interplay to regulate T cell activation (136). This greatly hampers the intended use of these methods in disease outcome predictions. With the rise in available TCR sequencing data offers the opportunity for researchers to improve methods of epitope prediction and specificity identification. Over the recent years, numerous TCR-antigen specificity predictions tools have been developed, including TCRMatch, NetTCR-2.0, Deep TCR, and TCRAI.

TCRMatch takes advantage of the ever-growing data available to researchers in the Immune Epitope Database (IEDB) (103) that have been experimentally determined to be recognized by T cells and have their specificity information available (137, 138). This tool takes the TCR β chain CDR3 sequences and matches it against the existing sequences in the IEDB to identify specificity of each hit. TCRMatch performs well on independent and small datasets of paired CDR3αβ sequences and is available as a web server tool. However, the performance TCRMatch is affected by the accuracy and limitations in the publicly available data.

NetTCR-2.0 was developed to specifically address the limitations associated with simpler sequenced based models (104). NetTCR-2.0 is utilizes a complex convolutional neural network (CNN) to predict TCR-pMHC interactions based on the amino acid sequences of the peptide and CDR3 region of the TCR chains. CNN is a deep learning method that extracts important features from sequenced data (105). The main advantage of CNNs is that it can learn sequence motifs through objective functions provided to the network. These motifs can be used by the deep learning model to either describe the data or to classify it. The 1D CNN model used in NetTCR-2.0 was found to outperform simpler sequence-based models such as TCRMatch and TCRdist (104). However, the accuracy of the CNN relied on being trained on paired TCRα- and β-chains. Due to the small number of training peptides, the model can only be applied to the limited set of peptides included in the training. NetTCR-2.1 is an extension of NetTCR-2.0 covering more peptides and all CDRs in the binding prediction (139).

DeepTCR is a platform for both supervised and unsupervised deep learning that can be applied at both the individual TCR level and repertoire level (105). The aim of this method is to learn patterns in data that can be used to describe or predict sequence motifs. However, this method also runs into the same problems as NetTCR due to the limitations in the training data.

TCRAI utilizes a similar neural network as DeepTCR, and both methods outperformed TCRdist and NetTCR (106). TCRAI was also more balanced in terms of sensitivity and specificity compared to GLIPH2, NetTCR, TCRdist and DeepTCR. The flexible architecture, ID convolutions, batch normalization of CDR3 sequences and lower dimensional representations for the genes forced TCRAI to learn stronger gene associations making it a stronger performer compared to its rival DeepTCR.

As constantly highlighted in the above methods, a major drawback of deep learning models is that they do not perform as well when tested on different datasets that do not belong to the same source as the training data (140). In order to make machine learning models applicable for real-world applications, they would need to be trained on larger-scale datasets while exploring different feature representations for unseen TCRs and/or epitopes (141).

## Identifying antigen specificity has applications in autoimmunity, allergy, cancer and infectious diseases

An individuals’ TCR repertoire is incredibly diverse, however conditions including autoimmune disease, allergy, cancer, and infections can lead to clonal expansion of antigen specific T cells. Using the methods described in this review, the TCR repertoires of clonally expanded cells can be studied in different disease states to better understand antigen specificity. Here, we review recent findings using TCR repertoires to define antigen-specific T cells.

### Autoimmunity and allergy

Autoimmune and allergic diseases are defined by a breakdown of tolerance. In the case of autoimmunity, antigen specific T cells bind to antigen presenting cells (APC) presenting self-peptides; whereas in allergy, they recognize APC presenting harmless environmental agents. The recognition of these antigens by T cells leads to downstream inflammatory cascades and results in numerous forms of disease affecting almost every part of the body. T cells are known to play a significant role in these diseases in their recognition of self-antigen followed by downstream activation of B cells and infiltration of tissues leading to immunopathology (142, 143). Many autoimmune diseases are linked to specific HLA genes, and the identification and characterization of clonally expanded T cells, by defining their TCR repertoire, and their respective antigens will enable us to better understand the development and pathogenesis and ultimately treat patients with these diseases (144). The major challenge with this has been the low abundance of these cells in circulation, however the advent of single cell technologies and paired transcriptome/TCR analyses has opened the door to new studies on these populations (145).

Studies have examined TCR repertoires in autoimmune diseases (144, 146) including Crohn’s Disease (147), systemic lupus erythematosus (SLE) (148, 149), rheumatoid arthritis (RA) (150–152), celiac disease (CD) (153, 154), type 1 diabetes (T1D) (155, 156), and Lofgren’s Syndrome (LS) (157). This large body of work shows that antigen specific T cells are critical for disease pathology, expand clonally during disease, can be tracked in tissue and blood, and have broad shifts in disease-specific TCR repertoires (144, 146). More recent studies have begun to comprehensively characterize these cells using paired single cell RNA-Seq and TCR sequencing. One study examined patients with psoriatic arthritis to show predominantly CD8+ clonal expansions in the joint fluid, pointing to a critical role for these cells in disease (158). Another study examined skin inflammatory diseases, finding differences in the transcriptional signatures and clonal expansion of T cells in psoriasis versus atopic dermatitis (159). A third study showed clonal expansion of activated, cytotoxic T cells in cerebrospinal fluid in patients with multiple sclerosis (MS) (160). In another study, TCRs expressing disease associated public β-chain variable region BV9–CDR3β motif were isolated from individuals with ankylosing spondylitis and acute anterior uveitis (161). Using HLA-B*27:05 yeast display peptide libraries, authors identified shared self-peptides and microbial peptides that activated T cells expressing both ankylosing spondylitis and acute anterior uveitis disease-specific TCRs. Their structural analysis revealed cross-reactivity to be rooted in shared binding motifs present in both self-antigens and microbial antigens that engages the BV9–CDR3β TCRs. More studies targeted at antigen-specific cells are needed, which is dependent on the discovery of antigens and epitopes associated with autoimmune diseases. Importantly, studies in mice have shown that TCR affinity plays a role in the activity of autoreactive T cells, making it critical for us to understand the strength of interactions between TCRs and their epitopes (162). Further, there is translational potential in targeting these antigen-specific cells for use in therapy by immune suppression (163, 164).

Allergic diseases encompass a wide range of pathologies, but are mediated by immune responses to environmental agents including aeroallergens and foods. While it is known that T cells play a role in allergy, specifically activated T helper type 2 (Th2) cells, the mechanisms leading to the break of tolerance and development of disease remain unknown. The identification and characterization of allergen-specific T cells, their TCR sequences, and their reactive epitopes is critical for our ability to better treat patients with allergies (145). Studies on various tissue compartments and blood have broadly shown skewing of T cell repertoire usage with allergic disease (165–169). More targeted studies have examined the TCR repertoires and transcriptional profiles of antigen-specific T cells. A study on dog-allergen specific T cells showed heterogeneity in Th2 cells along with less clonality in allergic individuals (170). Alternately, studies on peanut allergy have shown TCR convergence in antigen-specific cells from allergic patients (171, 172). A recent study on eosinophilic esophagitis (EoE) analyzed esophageal, peripheral blood, and duodenal samples, showing clonal expansion of a pathogenic effector Th2 (peTh2) population in these compartments with EoE (173). These studies suggest specific antigens elicit T cell responses in allergy, but require more studies defining and validating T cell epitopes in the allergens. Additionally, the significant heterogeneity in individual responses and in responses to different types of allergens require more studies examining the TCR repertoires of antigen-specific T cells in allergy and other diseases.

### Cancer

Cancer is a disease of the genome- cells that are unable to prevent or repair oncogenic mutations can metastasize and develop into tumors (174). The same mutations that drive oncogenesis generate peptides that can be processed and presented as epitopes on the surface of cancer and antigen presenting cells. Importantly, T cells can recognize said epitopes through their TCRs in order to initiate an anti-tumor immune response (175, 176). This phenomenon led to the development of T cell-based therapies for cancer. Adoptive cell therapy, T cell epitope vaccines, and immune checkpoint blockade therapy all aim to magnify the number of cancer-specific T cells in order to bolster a patient’s immune response. Unlike traditional cancer treatments (i.e. chemotherapy, radiation, etc.), T cell based cancer therapy can be designed to specifically target cancer cells and thus limit off-target toxicities (177). For this reason, much work has been done within the T cell field to identify cancer-specific epitopes (i.e., neoepitopes), neoepitope-specific T cells, and their respective TCRs. Many previous works have discussed current neoepitope prediction tools that harness tumor and blood sequencing to identify tumor-specific epitopes (178–182). In this section, we will highlight work that uses RNA and TCR sequencing to characterize tumor-specific T cells.

Many studies within this either infer or identify antigen specificity of T cells and characterize the phenotype of the predicted tumor-specific cells (183–185). Work done by Li et al. provides an example of this. Their study used single cell RNA and TCR sequencing of 25 melanoma patient tumors in order to investigate the T cell subsets present within tumors at different stages of the disease. It was observed that the T cells present in the tumors of these patients expressed genes associated with cell dysfunction. When the intratumoral cells were characterized in more detail, it was found that they are present in a spectrum of dysfunctionality: cells either expressed genes associated with transitional, early or high dysfunctionality. Importantly, the highly dysfunctional cells were exclusively present in the tumor of the patients and not in the peripheral blood. By including the clonality information gained through TCR sequencing, it was discovered that clone size was significantly increased in the dysfunctional cells that were exclusively present within the tumor. Thus, leading to the conclusion that these clonally expanded T cells that exhibit a highly dysfunctional gene program within the tumor are potentially tumor-specific (186). Other work has also classified tumor-specific cells as dysfunctional. In particular, recent work by Lowery et al. employed single cell RNA sequencing of 10 metastatic human tumors to generate a UMAP of 12 phenotypically distinct clusters of cells. This study also isolated neoantigen-specific T cells by culturing tumor infiltrating lymphocytes (TILs) from the same 10 patients with peptide or tandem-minigene pulsed dendritic cells and sorting for activated cells. Integrating the TCRs of the epitope specific cells onto the UMAP revealed that the majority of the epitope-specific cells congregated within the dysfunctional CD4 and CD8 phenotypic cluster. This led to the identification of a dysfunctional gene signature comprising 283 genes that were associated with the neoepitope-specific dysfunctional cells which, in turn, resulted in the identification of additional neoepitope-specific T cells (187). Lastly, Gros et al. found that PD-1 expression, a gene associated with dysfunction and exhaustion, could be used to narrow down the identification of cancer-specific T cells. PD-1 expression was found in 36% of the TIL isolated from 18 tumors while only 4% of peripheral cells expressed PD-1. Both CD8+PD1+ and CD8+PD1- populations were sorted from patient PBMC and expanded with neoepitope candidates, which showed that CD8+PD1- cells had limited reactivity in comparison to their PD1+ counterparts. Importantly, there was an overlap in the TCR sequences of CD8+PD1+ TILs and circulating cells but not in the PD1- population (188). This suggests that PD1+ cells within the tumor may be antigen-specific and that PD1 expression within the periphery may be circulating clones of the tumor-specific cells. Overall, this body of work provides an example of how antigen-specificity is employed to better understand the critical players within an anti-tumor immune response and develop concrete phenotypes, such as PD1 expression and/or dysfunctionality, of tumor-specificity.

Work has been done to characterize tumor-specific T cells outside of the expression of a dysfunctional phenotype and PD1. In particular, aspects of the TCR repertoire have been examined. For example, Reuben et al. studied the relationship between TCR repertoire overlap in the tumor tissue of 236 early-stage non-small cell lung cancer (NSCLC) patients and their adjacent uninvolved lung. Through TCR sequencing of the CDR3 β region in the tumor and adjacent lung, this group found an overlap of the TCR repertoire present within the tumor and adjacent uninvolved lung. Importantly, relapsed patients had a higher TCR repertoire overlap than non-relapse patients. This indicates that the presence of a larger repertoire of tumor-specific than shared T cell clones (i.e., less overlap) could be used as a prognostic marker for NSCLC patients (184).

Lastly, a few key studies have used the TCR sequence as a molecular barcode alongside single cell RNA sequencing to identify additional genes potentially relevant for tumor-specific cells. Zheng et al. used single cell RNA and TCR sequencing on tumor specific CD4 T cells in human melanoma. They found the TCRs of neoantigen-specific CD4 T cells and used this barcode to determine that these cells had significant expression of the genes HOPX and ADGRG1 and CXCL13 (189). Further, Pauken et al. characterized “tumor-matching” T cells in the peripheral blood (i.e., T cells in the blood expressing tumor-specific TCRs) as cells that expressed a more effector phenotype with a decreased expression of genes GYPC, CCR7, LTB, and FLT3LG (190). Overall, these provide an example of the work that has been done to utilize tumor-specific TCRs to identify additional markers outside the traditional exhausted and dysfunctional phenotype.

### Infectious disease

In addition to autoimmunity, allergy, and cancer, the TCR sequences of antigen-specific cells within the realm of infectious disease have also been investigated. This section will focus in particular on *Mycobacterium tuberculosis* (Mtb), Epstein-Barr Virus (EBV), and SARS-CoV-2.

Mtb is an infectious disease predicted to affect about ⅓ of the world’s population. Mtb is characterized by a spectrum of disease stats ranging from a latent, controlled version of infection (LTBI) to an active infection state (ATB) in which a person becomes contagious. A significant limitation in the effective treatment of this disease is the lack of effective diagnostic tools that can accurately identify individuals with LTBI who are at risk of developing ATB (191). For this reason, many groups have utilized TCR and RNA sequencing to study the repertoire of Mtb-specific T cells in order to get a more in depth understanding (192). Single cell TCR and RNA sequencing, calculating the frequencies of different TRBV, TRBD, and TRBJ comparing the tuberculosis pleural effusion (TPE) and blood in ATB patients revealed an increased expression of TRBV4-1 as well as genes related to TCR signaling, T cell activation, glycolysis and differentiation (193). Gideon et al. studied the role of different T cell subsets present within Mtb granulomas, a prominent feature of Mtb infection, in which immune activity can promote bacterial clearance or persistence. Single-cell RNA sequencing of granulomas derived from cynomolgus macaques infected with a low dose of Mtb revealed one particular cell cluster negatively correlated with bacterial burden - the T/NK cell cluster. This cluster (the so-called Type1-Type17 cluster) was enriched for a Th1 and Th17 phenotype, CD4, increased cytotoxic production, cytokines, and heat shock protein. However, these cells within this cluster were also enriched for common CDR3 sequences suggesting limited clonal expansion (194). Lastly, our own work characterizing the phenotype of antigen-specific cells using bulk RNA sequencing, revealed that HLA-DR expression is specific to recently divided Mtb-specific cells in ATB patients (195). However, additional work must be done to connect this phenotype to antigen-specific cells expressing specific TCRs.

The study of antigen-specific TCRs to characterize T cell responses is also applied to EBV and SARS-CoV-2 specific T cells. EBV is a gamma-herpesvirus that infects more than 80% of humans over the age of 20. EBV is known to infect B cells and EBV-specific immune responses are driven by T cells (196). EBV infection has been proven to precede multiple sclerosis onset (MS), therefore TCR sequencing has been used to analyze the TCR repertoire overlap in EBV and MS patients. Published antigen-specific TCRs derived from EBV, cytomegalovirus (CMV), influenza A, and SARS-CoV-2 were quantified in the blood of MS patients and MS-negative controls. This revealed a significantly larger number of EBV-specific TCRs in MS patients compared to healthy controls while none of the other infectious TCRs had a notable trend. Interestingly, MS patients that had undergone treatment that causes sequestration of T cells in the peripheral had an increase in EBV-specific T cells present. This indicates that there are EBV-specific cells creating an immune reaction within the CNS of MS patients that is removed upon treatment. The analysis of the transcriptome of EBV-specific T cells in MS patients and healthy controls determined that the T cells with EBV matching TCRs were enriched for an effector memory phenotype including the expression of PDCD1, CD28, KLRK1/NKG2D, TIGIT, NAM1, and CD244. Thus, this study identified and characterized EBV-specific T cells that may be implicated in MS symptom onset (197). Similar work has been done to study SARS-CoV-2. SARS-CoV-2 is the virus responsible for the COVID-19 pandemic and resulted in hundreds of thousands of deaths. Previous work has clearly shown the importance of T cell responses in COVID-19 related immunity and vaccination (198–201). For example, combining pMHC multimers to identify epitopes and TCR sequencing in a group of individuals with acute COVID-19 showed an enrichment of TRBV27 in epitope-specific T cells. These epitope-specific T cells were unable to produce cytokines and downregulated genes associated with activation, migration, and proliferation. Thus, Gangaev et al. was able to identify SARS-CoV-2 specific T cells and their overall phenotype, which gave insight into the characteristics of the antigen-specific T cells in acute disease (202).

## Antigen-specificity in unconventional T cells

Unconventional T cells are a relatively rare and understudied subset compared to canonical CD4 and CD8 T cells. Unconventional T cells are innate-like lymphocytes that have features of both innate and adaptive immune cells (203). They are not MHC-restricted like conventional T cells and are considered donor-unrestricted as they recognize monomorphic ligands that are shared across diverse human populations unlike MHC-restricted T cells. There are many unconventional T cell subsets, here we focus on mucosal-associated invariant T (MAIT), natural killer T (NKT), and γδT cells.

### MAIT cells

MAIT cells comprise only 2-5% of T cells in circulation and 10% of CD8+ T cells, but can be found at higher frequencies in tissues such as the liver (204, 205). Most MAIT cells express α-chain rearrangements with the genes TRAV1-2-TRAJ33/20/12 paired with a limited TCRβ-chain repertoire of Vβ2 or Vβ13 (TRBV6 or TRBV20, respectively). These pairings make up the vast majority of MAIT TCR clonotypes in circulation. MAIT cells recognize antigens presented by MR1, a non-polymorphic MHC I-like antigen-presenting molecule. There are other MR1-reactive T cell subsets described elsewhere (206), but classical TRAV1-2+ MAIT cells will be the main focus here.

MAIT cell antigens include those that are riboflavin-based, whereas MR1-restricted T cell antigens comprise a wide array of small molecules, which are reviewed elsewhere (206, 207). Riboflavin pathways are not present in mammals, so MAIT cells can respond to a broad array of microbially derived riboflavin intermediates. These are typically vitamin B metabolites derived from bacteria and yeast, of which the most frequently described and utilized is 5-(2-oxopropylideneamino)-6-D-ribitylaminouracil (5-OP-RU).

A common method to identify MAIT cells is through using MR1/5-OP-RU tetramers, which is sensitive and specific for this cell subset. However, CD4+ MAIT cells have an increased TCR diversity and only roughly one-third of this population binds to MR1/5-OP-RU tetramers (208).

### NKT cells

There are two main groups of NKT cells that can be separated based on the expression of specific TCRs and reactivity to different sets of antigens: Type I NKT and Type II NKT. Both NKT subsets recognize the antigen-presenting molecule CD1d, a monomorphic MHC class I-like molecule.

Type I NKT cells, also known as invariant NKT (iNKT) cells, is the more well-studied of the two NKT subsets. iNKT cells constitute roughly 0.1% of T cells in circulation and 1% of liver mononuclear cells in humans (205). iNKT cells have a single α-chain, TRAV10-TRAJ18, that pairs with a limited set of β-chains (209). The β-chain diversity dictates the antigen specificity of iNKT cells (209). iNKT cells recognize lipid antigens such as the prototypic iNKT antigen α-galactosylceramide (α-GalCer). Other iNKT cell antigens include other microbial glycolipids and self-lipids such as phosphatidylinositol (210).

Compared to iNKT cells, Type II NKT cells are more prominent in humans but are less well understood (211, 212). Type II NKT cells have a more diverse TCR repertoire. Type II NKT cells can recognize self, non-self, and non-self and non-microbial antigens (e.g., pollen) presented by CD1d (213). These antigens are largely either sphingolipids and glycerolipids or phospholipids, and include the self-lipid sulfatide identified in mice (214). Type II NKT cell TCRs can be specific to various antigens or promiscuous, i.e. different TCRs can recognize the same antigens (215).

Antigen recognition can be directly measured through the use of tetramers involving CD1d loaded with antigens of interest (216). CD1d tetramers loaded with αGalCer are typically used to identify iNKT cells (217). Sulfatide can also be loaded on CD1d to identify Type II NKT cells. Lipid-loaded CD1d tetramers have been utilized in numerous studies to identify reactive type II NKT cells in multiple diseases, including Type 1 Diabetes, Gaucher’s disease, and cancer (218). However, some antigens will be unable to form stable complexes with CD1d molecules, in which case tetramers cannot be used to identify reactive NKT cells.

### γδT cells

γδT cells express γδ TCRs instead of αβ TCRs that the other cell subsets discussed thus far express. γδT cells in total constitute roughly 5% of circulating T cells and up to 16% of T cells in tissues (219). γδT cells recognize viral, bacterial, tumor, and (stress-induced) self-antigens (220), but the antigens they recognize are not fully elucidated (219, 221). γδT cells are primarily segregated into different subsets based on the expression of one of eight δ chains, with Vδ1 and Vδ2 being the two most prominent subsets.

Vδ2 cells are the most prominent γδT cell subset in circulation and can make up 1-10% of T cells in the blood (222, 223). They predominantly express the Vγ9 chain, but can express other γ chains to a lesser extent (224, 225). Vγ9δ2 T cells typically represent roughly 4% of T cells in adult blood (205). They recognize phosphoantigens presented by butyrophilin molecules BTN3A1 and BTN2A1 (204, 205). The canonical antigen used to activate and expand Vγ9δ2 cells is (E)-4-hydroxy-3-methyl-but-2-enyl pyrophosphate (HMBPP) (226) or (E)-4-hydroxy-dimethylallyl pyrophosphate (HDMAPP) (227). These are intermediates of the non-mevalonate pathway and are used somewhat interchangeably. On the other hand, Vγ9-Vδ2+ cells do not respond to phosphoantigens, including HDMAPP (224). However, this subset has been found to clonally expand in response to CMV infection (228).

Vδ1 cells are less common in the blood but can be found more frequently in tissues such as the skin and mucosa. They can express a range of γ chains, and TCRγ chain usage is different at distinct locations within the body (229). Vδ1 cells recognize a variety of antigen-presenting molecules, including CD1b, CD1c, CD1d, and MR1 (221). The antigens recognized by Vδ1 cells are mainly lipids presented by CD1 molecules, though the melanoma-derived peptide MART-1 presented by HLA-A2 has also been found to be associated with Vδ1 response (230).

Other γδT cell subsets have also been studied albeit to a lesser extent. Vδ3, for example, has been shown to recognize MR1 independent of the antigen presented by the molecule (231). Additional Vδ3 antigens include annexin A2, a stress-induced ligand (221). There are even fewer studies on other γδT cell subsets, but there is some evidence of antigen-specific Vδ4 T cells in *S. aureus* infection and leukemia (232, 233).

Identifying antigen-specific γδT cells can be challenging because the antigens they recognize are not fully elucidated. Some of the difficulties in this field have been presented elsewhere (221), but these include that antigens could be derived from all groups of macromolecules (e.g., lipids, carbohydrates) and could be on the cell surface or in the extracellular space, both of which do not apply to canonical T cells. Similarly, TCR sequencing may not facilitate the identification of antigen-specific γδT cells due to these reasons. However, there has been progress in these efforts, including using tetramers with known antigens to identify reactive γδT cells (234) and unbiased biochemical screens to identify novel γδT cell antigens (235). Many studies have noted expression of specific markers associated with antigen recognition and γδTCR clonal expansion in numerous contexts, such as Mtb and HIV infection, which suggests antigen reactivity (219). Future work will continue contributing to our understanding of γδT cell antigen recognition and identification of antigen-specific cells.

## Concluding remarks

Measuring antigen-specific T cell responses and associated phenotypes helps to deepen our understanding of many different diseases. There is value in examining and understanding the repertoire of antigen-specific T cells, rather than focusing on individual epitopes or antigens. Isolating antigen-specificity allows researchers to better understand T cell biology in disease and ultimately to develop more targeted therapeutics and vaccines. Methods, highlighted in this review are utilized to study antigen-specificities and their associated phenotypes in a variety of contexts. However, there is a gap in these techniques and our knowledge to address issues of multi-epitope-specificity, and also MHC diversity and cross-reactivity. Thus, newer methods are constantly evolving surrounding this need and will continue to develop ushering in the next generation of tools better adapted to analyze complex repertoires and their responses to multiple epitopes.

## Author contributions

All authors contributed to the conceptualization, writing, editing and literature review. All authors contributed to the article and approved the submitted version.
